# Conversations About Children When an Important Adult Is at End of Life: An Audit

**DOI:** 10.1177/10499091211046241

**Published:** 2021-09-19

**Authors:** Jeffrey R. Hanna, Elizabeth Rapa, Mary Miller, Madeleine Turner, Louise J. Dalton

**Affiliations:** 1 Department of Psychiatry, University of Oxford, Warneford Hospital, Oxford, United Kingdom; 2 Department of Palliative Care, Sir Michael Sobell House, Oxford University Hospitals NHS Foundation Trust, Oxford, United Kingdom; 3 Nuffield Department of Medicine, University of Oxford, Oxford, United Kingdom

**Keywords:** end of life, children, dying, communication, healthcare professionals, social care professionals, family-centered care, psychosocial support

## Abstract

**Purpose:**

Health and social care professionals report it challenging to have conversations with families when an important adult in the life of a child is at end of life, often feeling this aspect of care is the responsibility of other colleagues. This study aimed to understand professionals’ perceived role in family-centered conversations as part of routine care at end of life, and how to promote this element of care in clinical practice.

**Methods:**

An audit was completed with 116 professionals who work in palliative care including doctors and nurses that attended a 2-day virtual congress.

**Results:**

Professionals (73.2%) felt confident about starting a conversation with adult patients at end of life about important children. However, enquiring about relationships with children was largely dependent on the age of the patient. 64.7% of respondents reported signposting families to websites and services that provide family support. Most professionals (76.7%) wanted training to equip them with the skills and confidence to having family-centered conversations at end of life, with videos demonstrating how to provide these elements of care the most preferred option.

**Conclusions:**

Short training resources should be developed to equip professionals with the necessary skills toward having conversations about children with patients and relatives in clinical appointments. There is a need for professionals to ask every patient about important relationships with children.

## Introduction

Global evidence has shown that effective communication with children about parental illness is essential for long term psychological wellbeing and family functioning.^1^ Health and social care professionals (hereafter referred to as professionals) are central to providing psychosocial support to families when an important adult in the lives of their children (<18 years old) is at end of life.^2-4^ Professionals often feel ill-equipped to have conversations with patients and their families about children; frequently reporting family-centered care to be the role of other colleagues within the multidisciplinary team.^2,5^ In reality, parents have reported a lack of supportive guidance from professionals about sharing information with their children about a significant adult’s illness, even when death is imminent.^6-8^ Professionals have reported a desire to develop their skills and confidence to have conversations in routine care with patients and their families about children from diagnosis through to end of life.^2,5,9,10^

One of the reported obstacles to conversations with patients about their children is professionals’ own discomfort with raising these sensitive topics.^2,3^ Professionals working in palliative care have extensive training, skills and experience talking with patients about illness, death and dying. The authors wanted to understand the extent to which these skills were used to consider the needs of children within the family. An audit was conducted with palliative care professionals to better understand: (1) professionals perceived role in having family-centered conversations with patients and families at end of life, and (2) clinicians’ views about support that would facilitate their provision of this psychological care to families about children in routine clinical practice.

## Methods

The audit consisted of an online open survey. This study is reported following the Checklist for Reporting Results of Internet E-Surveys (CHERRIES) criteria.^11^

### Audit Development

The audit was developed using JISC Online Survey software and tested by the research team. The questions were informed by a qualitative systematic review^3^ and 2 qualitative studies exploring professionals’ role in supporting families when a parent of dependent children is at end of life.^2,5^ The authors, who developed the audit, have a wealth of research and clinical experience in palliative care and communication with children regarding illness. The audit consisted of questions exploring professionals’ views about their role, confidence, and training needs to having family-centered conversations with families about important relationships with children. Professionals were also asked to state their professional role and years’ working in clinical as well as palliative care practice. The questions were not randomized, each question was presented on a new page, and respondents were unable to review and change their answers (e.g., through a “Back” button or a “Review” step at the end of survey) as this could have resulted in biased responses from respondents.

### Audit Pre-Testing

The audit was pre-tested with palliative care registrars, a palliative care nurse, and a palliative care consultant to check for its appropriateness, language, and understanding. Piloting the audit resulted in the rewording of some questions, the inclusion of additional criteria in the suggested responses, and the reordering of 2 questions. The decision was also made to highlight the questions as mandatory to complete the survey; the aim of the research was to have a better understanding of current practice and answering the 6 questions in the audit was considered necessary to enable this appreciation. The questions and response scale for each is reported in Table 1.

**Table 1.** The Questions and Response Criteria Used in the Survey.

Question	Responses
Question 1: Do you ask every patient “Do you have dependent children (under the age of 24 years)?”	Yes, it is a routine part of the care I offer to every patient. No as I know another colleague does this as part of their routine care. I judge whether to ask this question based on the patient’s age. No, as there are other more urgent priorities for my consultation with the patient.
Question 2: If dependent children are identified, do you ask the patient “What do the children know and understand about your illness?”	Yes, it is a routine part of the care I offer to every patient. No as I know another colleague does this as part of their routine care. I judge whether to ask this question based on the patient’s age. No, as there are other more urgent priorities for my consultation with the patient.
Question 3: If no dependent children are identified, do you still ask every patient “Do you have important relationships with any children (e.g., grandchildren, nephews and nieces)?”	Yes, it is a routine part of the care I offer to every patient. No as I know another colleague does this as part of their routine care. I judge whether to ask this question based on the patient’s age. No, as there are other more urgent priorities for my consultation with the patient.
Question 4: Do you offer guidance to patients and/or their support network about talking to children about the illness?	No—this is not requested by patients and/or their support network. No—I do not feel this is part of my role. No—I do not feel I have the specific skills to offer this guidance. No—there are other more urgent priorities for my consultation with the patient. No—this is the role of one of my colleagues in the team. Yes, if this is raised by patients or their support network. Yes, I make sure I raise this issue myself with every patient and/or members of their support network. *Supplementary to “Yes” options (presented on a new page)* How do you offer this guidance? I signpost to appropriate resources and websites. I discuss with patients how they can tell children about their illness.
Question 5: To what extent do you agree with the following statement: “I feel confident initiating discussions with a patient and/or their support network about how to talk to children about an important adult’s life-threatening illness?”	Strongly disagree Somewhat disagree Unsure Somewhat agree Strongly agree
Question 6: Would you like training on supporting patients to talk with important children about their illness?	Yes—I would like to acquire the knowledge and confidence to deliver this aspect of care. No—I do not feel the need for this training as there are already other professionals providing it. No—I feel I already have the skills and confidence to provide this aspect of care. Supplementary to “**YES**” option being selected (presented on a new page)‘Please prioritize your preferred method of training delivery this (1 being most preferred to 4 least preferred) – eLearning resources – Written materials such as leaflets and booklets – Videos that demonstrate how to have these conversations – Advanced communication skills training – Alternative suggestion(s) If you have selected “Alternative suggestion(s),” please provide your suggestion(s) below (free textbox provided)
Some questions about you	Professional role (drop down list): – Doctor—consultant – Senior Doctor (Non consultant) – Doctor in training – Community specialist nurse – Hospital specialist nurse – Team leader – Ward nurse – Ward sister – Occupational therapist – Physiotherapist – Chaplain – Other and please specify Length of time in healthcare practice: – <5 years – 5-10 – 11-15 – >16 Length of time in palliative care practice: – <3 years – 3-5 – 6-10 – 11-15 – >16

### Sample

The audit was distributed at the 2-day Palliative Care Congress virtual conference (host city; Edinburgh, United Kingdom) in March 2021. Individuals were considered eligible to complete the questions if they provide end of life care as part of their routine workload.

### Audit Administration

Individuals completed the audit by accessing the link in the research team’s exhibition stand. Prior to completing the questions, delegates were provided with information in the research team’s exhibition area about the purpose of the study, what their involvement would be, an estimate of how long the survey may take (up to 3 minutes), and the research team’s contact details to ask any questions. To encourage completion, delegates were informed they would be invited to enter a prize draw at the end of the audit, by clicking through to an unlinked form to provide their email address.

### Data Analysis

Data were analyzed by JRH using SPSS v.27 to provide details of the sample characteristics, and the frequencies and percentages of responses.

### Ethical Considerations

Delegates attending the virtual conference were not coerced to complete the audit. A member of the research team was “available” at the exhibition stand area during the 2-day conference to answer any queries or concerns of the delegates. Data protection procedures were observed. The research team provided a resource pack for all interested and participating respondents providing them with information about the purpose of the audit, information of organizations that provide family or professional support, the authors contact details, and links to psychoeducational resources designed for clinicians on how to support families in relation to dependent children about a parental life-threatening illness. Following consultation with the Joint Research Classification Group at University of Oxford, ethical approval was not required.

## Results

### Response Rate

The conference was attended by 588 professionals. Of these, 158 delegates visited the exhibition stand over the 2-day period. A total of 116 professionals entered and completed the survey, including doctors (n = 87) and nurses (n = 26), as well as a physiotherapist, pharmacist, and social worker. 88.7% of the sample had 5 or more years’ experience working in healthcare; 55.1% of the sample had at least 5 years working in palliative care. Professionals were based in the United Kingdom and Ireland. Sample characteristics are reported in Table 2.

**Table 2.** Characteristics of the 116 Respondents That Completed the Survey.

Professional role	*n*	Professional experience (years)	*n*	Palliative care experience (years)	*n*
Doctor (consultant)	38	<5 years	13	<3 years	39
Senior doctor (non-consultant)	10	5-10 years	32	3-5 years	13
Doctor (in training)	38	11-15 years	27	6-10 years	21
Junior doctor	1	>16 years	44	11-15 years	14
Community specialist nurse	3			>16 years	29
Hospital specialist nurse	8				
Palliative care nurse consultant	1				
Team leader	4				
Critical care nurse	1				
Ward nurse	5				
Ward sister	4				
Physiotherapist	1				
Pharmacist	1				
Social worker	1				

### Identifying Important Relationships With Children

Over a third of respondents (40/116) reported it is a routine part of their practice to ask patients if they have dependent children. This contrasts with over 50% of the sample (62/116) that decide to ask a patient if they have dependent children based on the patient’s age. When no dependent children are mentioned by patients, most professionals (84/116) stated they do not ask the patient about any other important relationships with children (Table 3).

**Table 3.** Identifying Important Relationships With the Children.

Question	Yes, it is a routine part of the care I offer to every patient	No, as I know another colleague does this as part of their routine care	I judge whether to ask this question based on the patient’s age	No, as there are other more urgent priorities for my consultation with the patient
Do you ask every patient “Do you have dependent children (under the age of 24 years)?”	40 (34.5%)	12 (10.3%)	62 (53.4%)	2 (1.8%)
If dependent children are identified, do you ask the patient “What do the children know and understand about your illness?”	81 (69.8%)	11 (9.5%)	13 (11.2%)	11 (9.5%)
If NO dependent children are mentioned, do you still ask every patient “Do you have important relationships with any children (e.g. grandchildren, nephews and nieces)?”	32 (27.6%)	12 (10.3%)	29 (25%)	43 (37.1%)

### Providing Psychosocial Care to Families About the Children

Less than a quarter of professionals (29/116) stated they raise the issue of how to talk to the children with every patient or a member of the family. Most respondents (62/116) reported that when the issue has been raised by the patients or their support network, they provide guidance to patients and/or their support networks about talking to their children about the illness (Table 4). Many respondents (64.7%) considered it to be their role to signpost patients and their support networks to appropriate resources and websites that provide family support, advice, and guidance on telling children about an illness.

**Table 4.** Perceptions of Providing Psychosocial Care to Families About the Children in Routine Care.

Question	No—this is not requested by patients and/or their support network	No-I do not feel this is part of my role	No, I do not feel I have the specific skills to offer this guidance	No—this is the role of one of my colleagues in the team	Yes—if this is raised by patients or their support network	Yes—I make sure I raise this issue myself with every patient and/or members of their support network
Do you offer guidance to patients and/or their support network about talking to children about the illness?	2 (1.8%)	1 (0.9%)	15 (12.9%)	7 (6%)	62 (53.4%)	29 (25%)

### Confidence and Skills to Deliver Family-Centered Care

While the majority of the professionals (73.2%) somewhat or strongly agreed they are confident initiating discussions about children (Table 5), 76.7% of respondents still reported a desire for training to deliver this aspect of care (Table 6). Professionals reported their preferred method of training would be videos demonstrating how to have conversations with patients about children (74/116; 63.8%), followed by classroom-based advanced communication skills training (67/116; 57.8%). Written materials such as leaflets and booklets were the least preferred choice for the provision of training (9/116; 7.8%).

**Table 5.** Professionals’ Confidence in Initiating Discussions With Patients About the Children.

Question	Strongly disagree	Somewhat disagree	Unsure	Somewhat agree	Strongly agree
To what extent do you agree with the following statement: “I feel confident initiating discussions with a patient and/or their support network about how to talk to children about an important adult’s life-threatening illness?”	9 (7.8%)	16 (13.8%)	6 (5.2%)	64 (55.2%)	21 (18%)

**Table 6.** Professionals’ Views on Training About How to Support Patients to Talk to Children About Their Illness.

Question	Yes—I would like to acquire the knowledge and confidence to deliver this aspect of care	No—I do not feel the need for this training as there are already other professionals providing it	No—I feel I already have the skills and confidence to provide this aspect of care
Would you like training on supporting patients to talk with important children about their illness?	89 (76.7%)	9 (7.8%)	18 (15.5%)

## Discussion

There remains a critical gap in the provision of psychological care to families at end of life regarding the important relationships a patient has with children. Strikingly, the majority of professionals make a decision about whether to ask patients at end of life about relationships with children based on their age. This potentially overlooks children (e.g., grandchildren, nieces, nephews) who have a significant relationship with patients and may need support to understand their loved-one’s illness and death.

Most respondents felt confident about initiating conversations with patients at end of life about children, and described their involvement as signposting patients and families to websites and services that provide family support. A similar finding had been reported in the literature.^2,3,7,10^ However, the majority of professionals reported they want training to equip them with the skills and confidence to provide families with psychosocial support at end of life about children. Although evidence-based guidelines to scaffold raising these topics with patients are available,^1,12^ participants rated videos demonstrating how to deliver this aspect of care as their most preferred option for training (63.8%) followed by classroom-based courses (57.8%). There is a need to develop these specific resources, which would also be relevant to professionals working in many different medical specialties, with evidence indicating the benefits of raising these issues with patients earlier in their illness trajectory.^2,7^

### Implications for Practice

One of the greatest challenges faced by families is how best to prepare and support their children for the death of an important adult.^6,7,13,14^ Parents’ need support and guidance from professionals on how to: (1) tell children someone important to them has a serious illness and is going to die, and (2) prepare children for the actual death.^15,16^ Children who are prepared for a death are reported to cope and adjust better in bereavement with less input from psychological services, compared to children who are unprepared for a death of an important adult.^17,18^ Alongside this, children want to be informed of the reality of an important adult’s illness and involved in the end of life experience.^4,19^ Healthcare teams are removed from the emotional tension within the family which places them in a position to encourage parents to “start the conversation” with the children about the reality of a patient’s illness.^2,15^ Talking about important relationships with children in clinical appointments offers families the permission to put children on the agenda.

Psychosocial support in relation to dependent children should be incorporated into all routine appointments in adult services, highlighting family-centered care is the clinical responsibility of all professionals, similar to that of Scandinavian countries.^19^ To promote family-centered conversations in routine practice, it is important for professionals to ask all patients *“do you have important relationships with children?”*^9^ Professionals should be reassured that while conversations about children can be upsetting for families and for clinicians themselves, bereaved relatives have reflected it would have been if the healthcare team had initiated a discussion about children’s needs before the death happened and encouraged families to involve the children in the end of life experience.^7,9,20^ Additionally, bereaved families describe the limitations of leaflets and websites about how to talk to children about illness and death. Rather, families want professionals to start the conversation with them (the patient /partner) about how to navigate the end of life experience with their children.^7^ Professionals should be reassured that family-centered conversations do not need to be arduous or time consuming; short training resources such as video tutorials could equip clinicians with the skills to support the provision of family-centered care in practice.

### Strengths and Limitations of the Study

While it may be argued a biased sample of professionals were included in this audit (respondents were attending a palliative care conference), findings highlight a need for training to support clinicians’ provision of family-centered care in routine practice. Findings represent a range of professionals providing end of life care throughout the United Kingdom and Ireland. The survey did not ask specific questions about professionals’ role in providing psychosocial support to families from minoritised groups; this should be investigated through future research.

## Conclusion

Despite palliative care professionals’ advanced communication skills training, there remains a desire for further training to support and guide staff in talking to patients about the effect of the illness on children in their family. Professionals feels these skills would be best developed through specific video resources.
